# Longitudinal Study of Primary HIV-1 Isolates in Drug-Naïve Individuals Reveals the Emergence of Variants Sensitive to Anti-HIV-1 Monoclonal Antibodies

**DOI:** 10.1371/journal.pone.0017253

**Published:** 2011-02-23

**Authors:** Bijayesh Haldar, Sherri Burda, Constance Williams, Leo Heyndrickx, Guido Vanham, Miroslaw K. Gorny, Phillipe Nyambi

**Affiliations:** 1 Department of Pathology, New York University School of Medicine, New York, New York, United States of America; 2 Veterans Affairs New York Harbor Healthcare System, New York, New York, United States of America; 3 Department of Microbiology, Institute of Tropical Medicine, Antwerp, Belgium; 4 Department of Biomedical Sciences, University of Antwerp, Antwerp, Belgium; Massachusetts General Hospital, United States of America

## Abstract

To study how virus evolution affects neutralization sensitivity and to determine changes that occur in and around epitopes, we tested the ability of 13 anti-HIV-1 gp120 (anti-V2, anti-V3, anti-CD4bd and anti-carbohydrate) human monoclonal antibodies (mAbs) to neutralize sequential viruses obtained from five HIV-1 chronically infected drug naïve individuals. Overall, primary viruses collected from patients at first visit were resistant to neutralization by all anti-HIV-1 mAbs with the exception of one virus sensitive to IgG1b12. Four of the five patients' viruses evolved increased sensitivity to neutralization by anti-V3 mAbs. Virus collected from a patient obtained 31 months later, evolved increased sensitivity to anti-V2, anti-V3, and anti-CD4bd mAbs. Furthermore, the anti-V2 and anti-CD4bd mAbs also exhibited increased neutralization capacities against virus collected from a patient 29 months later. Of the seven anti-V3 mAbs, five showed increased potency to neutralize the evolved virus from a patient collected after 11 months, and three exhibited increased potency against viruses from two patients collected 29 and 36 months later. Anti-V3 mAbs exhibited the most breadth and potency in neutralizing the evolving viruses. Sequence analysis of the envelope regions revealed amino acid conservation within the V3 loop, while most of the changes identified occurred outside the core epitopes and in particular within the C3 region; these may account for increased neutralization sensitivity. These studies demonstrate that *in vivo*, HIV-1 can evolve increased neutralization sensitivity to mAbs and that the spectrum of neutralization capacities by mAbs can be broader when studied in longitudinal analysis.

## Introduction

To date, almost all studies that have examined the sensitivity of human immunodeficiency virus type 1 (HIV-1) isolates to anti-HIV-1 human monoclonal antibodies (mAbs) have been cross-sectional and they show that only a few mAbs neutralize HIV-1 primary isolates. The lack of neutralization by most antibodies has been attributed to a variety of reasons such as the occlusion of the neutralization sensitive epitopes on primary HIV-1 isolates by carbohydrate moieties, the absence of the specific epitope on the intact HIV-1 virion and the differences in conformational structures on the virions [1], [2]. In addition to these factors that contribute to neutralization resistance, HIV-1 primary isolates can evolve over time to escape from autologous neutralization through changes due to insertions and deletions, point mutations, changes in glycan shielding and nonsynonymous changes [3], [4], [5], [6], [7], [8].

HIV-1 viral envelope regions, which have been identified to be immunogenic and to which mAbs were developed include the V1V2, V3, CD4-binding domain (CD4bd), CD4 induced antigen (CD4i) and C5 regions of the gp120 [9]. The cluster I and II regions of the gp41 have also been found to be immunogenic to many mAbs [10], [11], [12]. Among the mAbs derived from the cells of HIV-1 infected individuals and that neutralize a variety of primary HIV-1 isolates in cross sectional studies, some are specific to epitopes in the V3 loop (e.g. 447-52D), CD4bd (e.g. IgG1b12), carbohydrate (e.g. 2G12), and others to epitopes in the MPER of gp41 (e.g. 2F5 and 4E10) [13], [14], [15], [16]. Antibodies directed at epitopes in the V1 region are known to be type specific while mAbs to V2 are cross-reactive but weakly neutralizing [17], [18]. Although some lab strains (e.g. MN) or extensively *in-vitro* cultured isolates (e.g SF162) are sensitive to neutralization by mAbs, many of these antibodies do not neutralize HIV-1 primary isolates when tested in cross sectional studies. However, several immunochemical studies have revealed that many of these antibodies, especially anti-V3 mAbs, bind to peptides, soluble proteins, recombinant proteins and intact virions, suggesting that the epitopes are present but in different forms [19].

Information is sparse on the evolution of sensitivity to neutralization of HIV-1 primary isolates by antibodies that either do or don't neutralize viruses tested in cross sectional studies. It is well known that viruses in infected individuals evolve to escape from neutralization by autologous antibodies over time [6], [8], [20], [21], [22]. No published study has demonstrated a scenario whereby viruses in HIV-1 infected individuals evolve increase sensitivity to their autologous antibodies. Instead, more virus diversification and escape from neutralization is documented [8]. Casting this in the vaccine context raises the issue of the relevance of virus neutralization sensitivity to heterologous antibodies and virus evolution.

The current study examines the neutralization sensitivity to anti-HIV-1 mAbs of viruses from 3 patients (ITM60, ITM27 and ITM39) [23] as well as 2 other patients (NYU104 and 3506 [unpublished]), whose viruses were previously tested with plasma to determine their neutralization sensitivity, and were shown to exhibit increased neutralization sensitivity (ITM60, NYU104, and 3506 [unpublished]), no change in neutralization sensitivity (ITM39), and decreased neutralization sensitivity (ITM27) [23]. Because the antibodies present in plasma are polyclonal, the use of anti-HIV-1 mAbs directed at specific epitopes on HIV-1 virions provides the opportunity to identify the specific epitopes that exhibit the change in the neutralization patterns seen with the polyclonal antibodies in plasma. Thus, in the present study, we examined the neutralization sensitivity of the sequential HIV-1 primary isolates during their natural evolution in HIV-1 infected drug naïve individuals to anti-HIV-1 mAbs directed at epitopes in the V2, V3, CD4bd and carbohydrates of gp120.

## Results

### CD4 T cell profiles of study subjects

**Figure 1:**
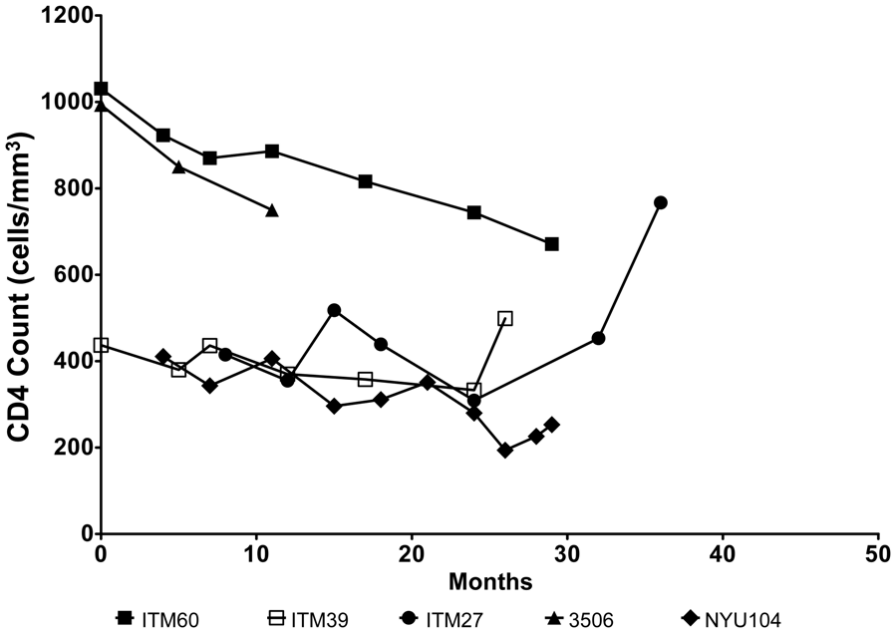
CD4 profile of HIV-1 infected study subjects. All the study subjects were asymptomatic. CD4 counts were determined by FACScan.

A portion of the sequential blood samples were collected from the five HIV-1-infected subjects and used to determine the CD4 cell counts by FACScan. Their CD4 profiles are shown in figure 1 and reveal that all these subjects studied were asymptomatic during the study period and were naive to antiretroviral drugs. At the start of the study, the CD4 counts of three study subjects (ITM27, ITM39, and NYU104) ranged between 411 and 437 cells/mm^3^, while the CD4 counts of two study subjects (ITM60 and 3506) was 1031 and 993 cells/mm^3^. The CD4 counts of ITM60 and NYU104 declined to 671 and 253 cells/mm3, while the CD4 counts of two study subjects (ITM39 and 3506) stayed relatively stable over time (499 and 750 cells/mm^3^), respectively. It was noted that the CD4 T cell count of subject ITM27 increased from 415 to 767 cells/mm^3^. Viruses isolated from a portion of the blood sample that was used for CD4 count determination were used for the neutralization studies described below.

### Neutralization by anti-V3 mAbs

**Figure 2:**
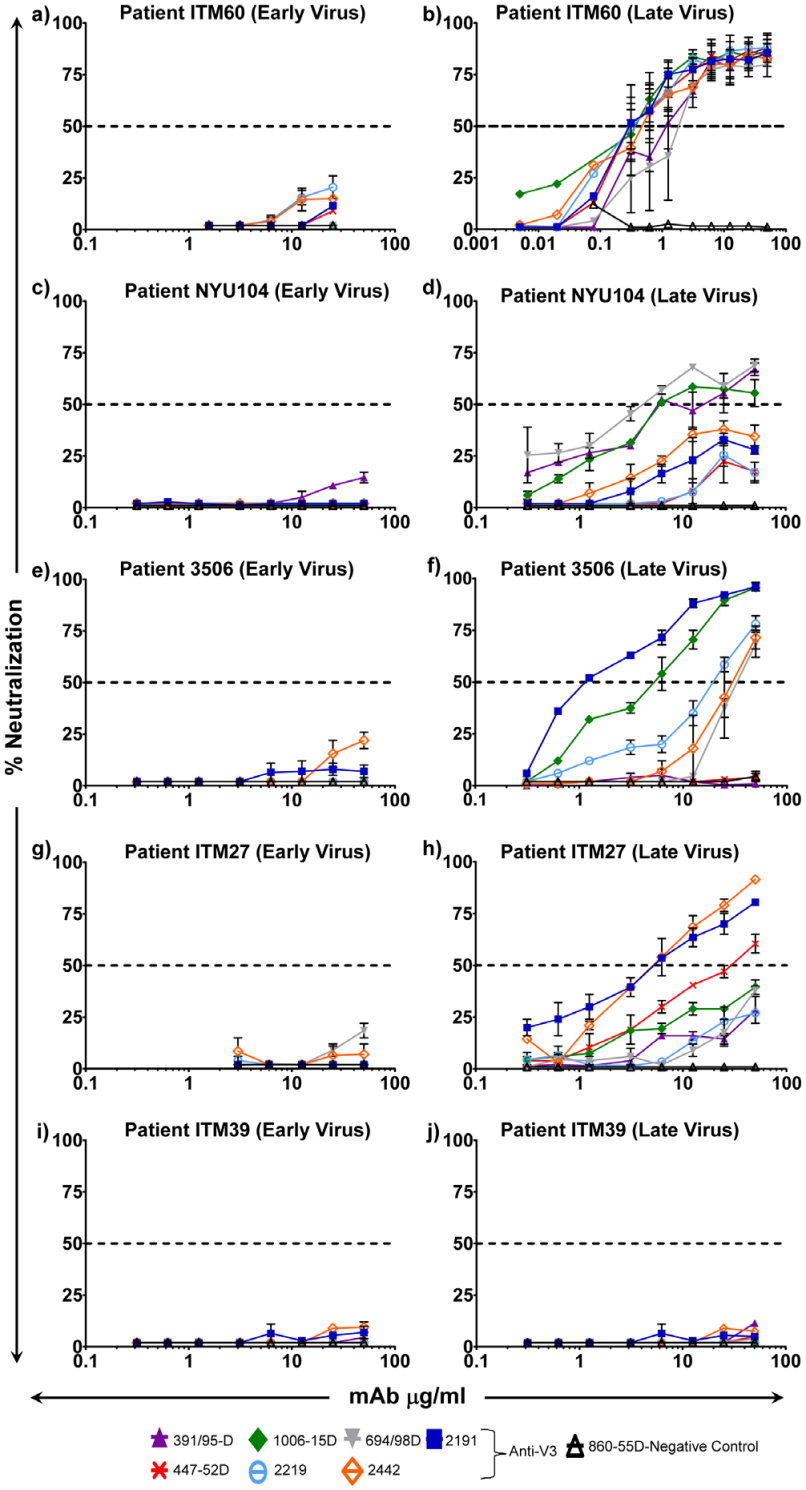
Neutralization curves of anti-V3 monoclonal antibodies against sequential viruses. Neutralization sensitivity of HIV-1 subtype B and CRF02_AG viruses obtained sequentially from patient ITM60 (a and b), NYU104 (c and d), 3506 (e and f), ITM27 (g and h), and ITM39 (i and j) with anti-V3 monoclonal antibodies. The dash horizontal line represents 50% neutralization.

Each of the seven anti-V3 mAbs, including 391/95-D, 447-52D, 1006-15D, 2219, 694/98-D, 2442 and 2191, were serially diluted at a range of 50-0.005 µg/ml and tested with the two sequential viruses isolated at early and late time point from patient ITM60, ITM27, ITM39 and 3506 infected with HIV-1 subtype B strain as well as NYU104 infected with a CRF02_AG variant. None of these mAbs neutralized the *early* virus from these patients above 50% (Figures 2a, 2c, 2e, 2g and 2i). All seven mAbs neutralized the later time point virus of ITM 60 (Figure 2b), with a capacity as high as 88% (Figure 2b). The lowest concentration of mAb needed for 50% neutralization (IC_50_) of the later time point virus was 0.3 µg/ml, as shown by mAb 447-52D and 2191 (Table 1) and the highest IC_50_ was 2.1 µg/ml for mAb 694/98-D (Table 1). Three mAbs (694/98-D, 391/95-D and 1006-15D) exhibited >50% neutralization of the later time point virus of NYU104. The specific IC_50s_ required for 50% neutralization were, 5.9 µg/ml for mAb 391/95-D, 6.1 µg/ml for mAb 1006-15D, and 4.4 µg/ml for mAb 694/98-D (Table 1). This data suggests that non-B subtype viruses can also evolve increased neutralization sensitivity to clade B derived mAbs. Five mAbs exhibited ≥50% neutralization at different concentrations against the virus taken 11 months later from patient 3506 (Figure 2f and Table 1). Of these five mAbs, 2191 exhibited an IC_50_ of 1.5 µg/ml (Figure 2f and Table 1) and an IC_90_ of 18.8 µg/ml (Figure 2f). Another mAb, 1006-15D, exhibited an IC_50_ of 5.5 µg/ml (Figure 2f and Table 1) and an IC_90_ of 27.1 µg/ml (Figure 2f). While mAb 2219 exhibited an IC_50_ of 20.5 µg/ml, the other two mAbs, 2442 and 694/98-D exhibited an IC_50_ of 31.5 µg/ml and 35.1 µg/ml, respectively (Table 1). Three of the seven anti-V3 mAbs neutralized (>50%) virus isolated 36 months later from patient ITM27 (Figure 2h) but none of the mAbs neutralized the late virus from patient ITM39 (Figure 2j). The most potent of the seven mAbs tested against the late virus from patient ITM27 was mAb 2442 with an IC_50_ at 5.4 µg/ml and an IC_90_ of 47.5 µg/ml (Table 1). Furthermore, IC_50_ was also achieved with mAb 2191 at a concentration of 5.5 µg/ml and mAb 447-52D at 30.6 µg/ml (Table 1).

**Table 1:** Anti-HIV-1 monoclonal antibody concentrations (µg/ml) required to yield 50% neutralization with the HIV-1 infected patients' sequential viruses.

		Patient Virus									
		ITM60		NYU104		3506		ITM27		ITM39	
Monoclonal Antibody		Early	Late	Early	Late	Early	Late	Early	Late	Early	Late
	391/95-D	>50	1.1	>50	5.9	>50	>50	>50	>50	>50	>50
	447-52D	>50	0.3	>50	>50	>50	>50	>50	30.6	>50	>50
	1006-15D	>50	0.4	>50	6.1	>50	5.5	>50	>50	>50	>50
Anti-V3	2219	>50	0.3	>50	>50	>50	20.5	>50	>50	>50	>50
	694/98-D	>50	2.0	>50	4.4	>50	35.1	>50	>50	>50	>50
	2442	>50	0.5	>50	>50	>50	31.5	>50	5.4	>50	>50
	2191	>50	0.3	>50	>50	>50	1.5	>50	5.5	>50	>50
	697-D	>50	37.7	>50	50	>50	>50	>50	>50	>50	>50
Anti-V2	2158	>50	21.4	>50	24.3	>50	>50	>50	>50	>50	>50
	559/64-D	>50	0.5	>50	10.1	>50	>50	>50	>50	>50	>50
Anti-CD4bd	654-D	>50	<0.3	>50	>50	>50	>50	>50	>50	>50	>50
	IgG1b12	>50	<0.3	>50	10.5	6.0	0.4	>50	>50	>50	>50
Anti-Carbohydrate	2G12	>50	>50	>50	3.7	>50	>50	>50	>50	>50	>50

Taken together, these data suggest that the viruses isolated at a later time point from patients ITM60 showed increased neutralization sensitivity to all the 7 anti-V3 mAbs tested, that from NYU104, ITM27 and 3506 showed increased neutralization sensitivity to 3–5 out of 7 anti-V3 mAbs, whereas the late time point virus isolated from ITM39 was resistant to neutralization by all the 7 anti-V3 mAbs as its early time point virus.

### Neutralization by anti-V2, anti-CD4bd and anti-carbohydrate mAbs

**Figure 3:**
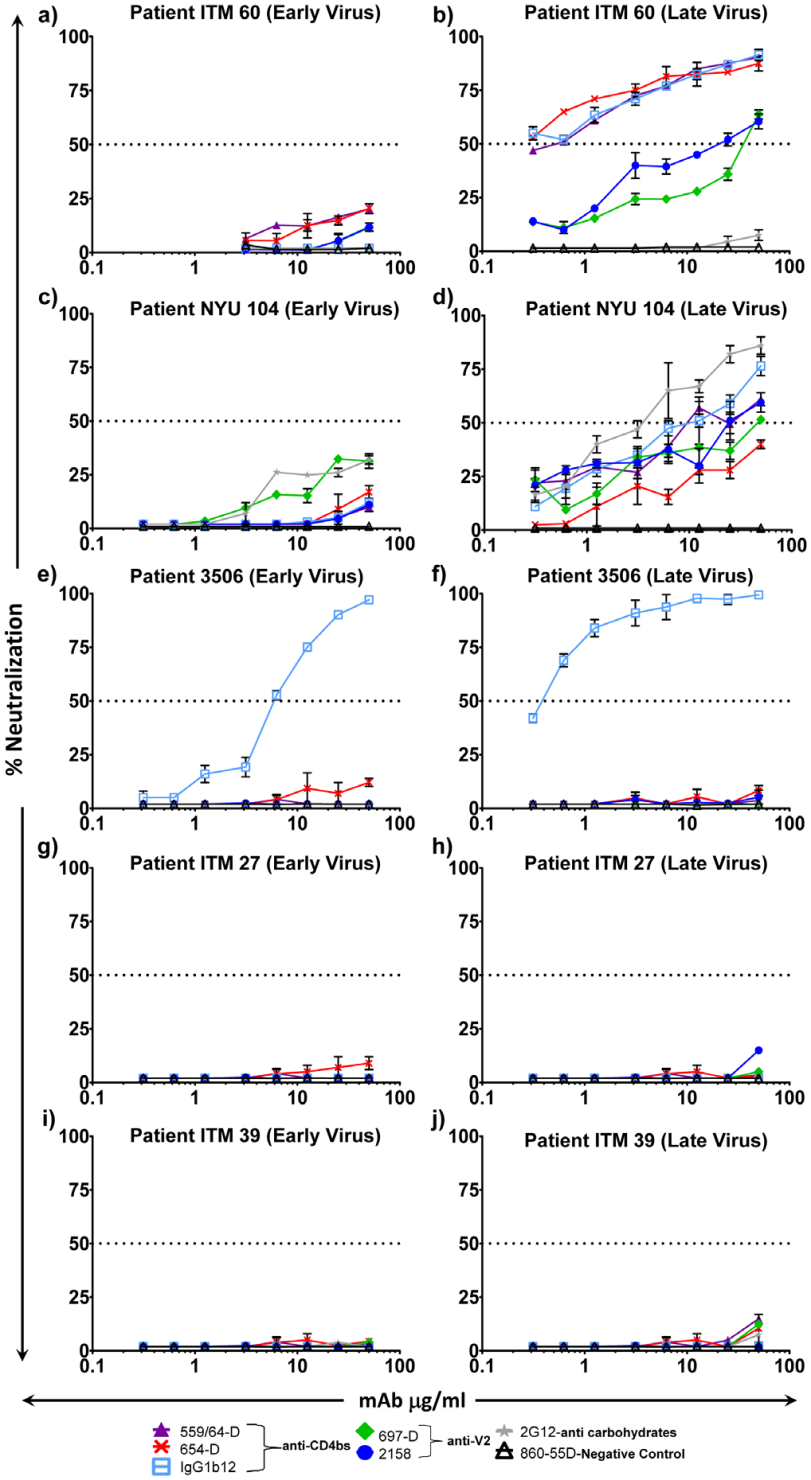
Neutralization curves of anti-V2, anti-CD4bd, and anti-carbohydrate monoclonal antibodies against sequential viruses. Neutralization sensitivity of HIV-1 subtype B and CRF02_AG viruses obtained from patient ITM60 (a and b), NYU104 (c and d), 3506 (e and f), ITM27 (g and h), and ITM39 (i and j) with anti-V2, anti-CD4bd, and anti-carbohydrate monoclonal antibodies. The dash horizontal line represents 50% neutralization.

We examined the neutralization patterns of two anti-V2 mAbs (697-D and 2158), three anti-CD4bd mAbs (559/64-D, 654-D and IgG1b12) and one anti-carbohydrate mAb (2G12), with the sequential viruses from all five patients; ITM60 (Figures 3a and 3b), NYU104 (Figures 3c and 3d), 3506 (Figures 3e and 3f), ITM27 (Figures 3g and 3h) and ITM39 (Figures 3i and 3j). While none of the mAbs neutralized the early viruses, with the exception of IgG1b12 which neutralized virus from patient 3506, extensive neutralization was achieved against the late time point viruses from patients ITM60 and NYU104 (Figures 3b and 3d).

As shown in Figure 3b, the pattern of neutralization exhibited by the three anti-CD4bd mAbs against the late virus from patient ITM60 was similar when tested at concentrations ranging between 0.3–50 µg/ml. As noted for mAb IgG1b12, IC_50_ and IC_90_ were achieved at concentrations of <0.3 µg/ml and 41.7 µg/ml, respectively. With mAb 654-D, an IC_50_ was also achieved at a concentration of <0.3 µg/ml (Table 1). For mAb 559/64-D, IC_50_ and IC_90_ were achieved at concentrations of 0.6 µg/ml and 45.8 µg/ml, respectively (Figure 3b and Table 1). For the two anti-V2 mAbs tested, the IC_50_ for 2158 was 21.4 µg/ml and that for 697-D was 37.7 µg/ml (Figure 3b, Table 1). Thus, for all three anti-CD4bd mAbs, IC_50_ was achieved at much lower concentrations compared to the anti-V2 mAbs.

Of the three anti-CD4bd mAbs (559/64-D, 654-D and IgG1b12) tested against the late virus from patient NYU104, mAbs 559/64-D and IgG1b12 neutralized the virus with IC_50_ of 10.1 µg/ml and 10.5 µg/ml, respectively (Figure 3d, Table 1). For the two anti-V2 mAbs tested, the IC_50_ for 2158 was 24.3 µg/ml and that for 697-D was 50 µg/ml (Figure 3d, Table 1). Interestingly, the late time point virus for NYU104 evolved increased neutralization sensitivity to the anti-carbohydrate mAb, 2G12, with an IC_50_ of 3.7 µg/ml (Figure 3d, Table 1).

All the mAbs described above were also tested with the sequential viruses from patient 3506. As shown in Figures 3e and 3f, IgG1b12 neutralized both early and late viruses from patient 3506, though exhibiting a higher neutralizing capacity against the late virus. Specifically, the IC_50_ and IC_90_ titers for mAb IgG1b12 were achieved at 6.0 µg/ml and 24.9 µg/ml respectively for the first time point virus (Figure 3e and Table 1), 0.4 µg/ml and 2.9 µg/ml respectively for the later time point virus, thus showing a higher potency of neutralization of the later time point virus (Figure 3f and Table 1).

All the mAbs described above were also tested with the sequential viruses from patient ITM27 and ITM39. None of the mAb neutralized the early and late viruses from patient ITM27 (Figures 3g and 3h) and ITM39 (Figures 3i and 3j).

### Examination of the presence of core epitopes on the sequential viruses targeted by the mAbs

We examined the gp120 sequences to determine if changes occurred within or outside defined core epitopes on the viral envelopes for those viruses that exhibit changes in neutralization sensitivity. Using the aliquots of the virus that were tested in the neutralization studies, we sequenced the viral envelope regions and analyzed the mutational changes by comparing the sequences from the initial viral isolates with those from the later time points.

### V3 epitopes

**Figure 4:**
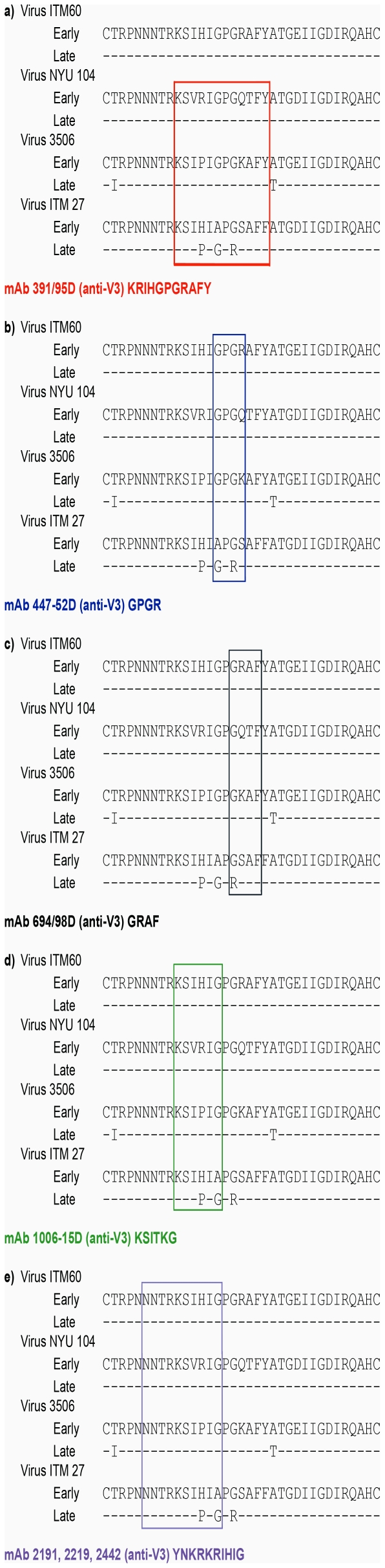
V3 loop sequences of the sequential viruses. The sequences shown represent the consensus sequence from 3-11 clones. The sequences were derived from the same aliquot of virus culture supernatant that was used in the neutralization assay. The core epitope to which the anti-HIV-1 monoclonal antibodies were directed is boxed. The epitope specificity of the monoclonal antibodies (mAb) was mapped using HIV-1 MN V3 peptides and the core epitope sequences of the MN peptide are shown along with the monoclonal antibody.

As shown in Figure 4, the V3 sequences of the sequential viruses from patients ITM60, NYU104, 3506 and ITM27, which evolved to become sensitive to anti-V3 mAbs, were examined. The lengths of the V3 loops of the sequential viruses from all four patients were the same (35 amino acids). Overall, the V3 sequences were fairly constant over time, with only a few mutations noted in the later time point virus compared with the virus from the first visit (Figure 4).

**Figure 5:**
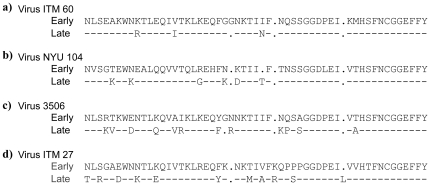
C3 region sequences of the sequential viruses. The sequences shown represent the consensus sequence from 3–11 clones. The sequences were derived from the same aliquot of virus culture supernatant that was used in the neutralization assay.

The V3 sequence used to define the mAb core epitope was derived from HIV-1 MN strain (www.hiv.lanl.gov) and the epitopes targeted by each mAb are shown in Figure 4. We compared the sequence of the HIV-1 MN-defined core epitope to the sequence of the epitopes on the sequential viruses studied, and correlated any differences in the sequence with neutralization sensitivity to the corresponding mAb. The most significant changes in the virus neutralization sensitivity to anti-V3 mAbs were noted with the sequential viruses from patients ITM60 and 3506 (Figures 2b and 2f). With the exception of one patient (ITM27), the core V3 sequence of the late time point viruses from all other patients was similar to that of the first time point virus (Figure 4, boxed). We then examined the sequences flanking the V3 loop, including the C2 and C3 regions, and found that mutations had occurred in these regions (data not shown and Figure 5a). The C3 region of the later time point virus from patient NYU104 that exhibited an increase in sensitivity to three of the seven anti-V3 mAbs as well showed several amino acid changes (Figure 5b). These results suggest that changes outside the V3 loop might also have contributed to the increase in neutralization sensitivity to anti-V3 mAbs.

The sequential viruses from patient 3506 exhibited an increase in sensitivity to five of the seven anti-V3 mAbs (Figure 2f). While the core epitope was similar to that of the initial virus, two changes had occurred outside the core V3 epitope in the virus obtained 11 months later. The two mutations included a change from threonine (T) to isoleucine (I) at position 2, and a change from alanine (A) to threonine (T) at position 22 (Figure 4). The change from hydrophilic threonine (T) to hydrobhobic isoleucine (I) at the base of the V3 loop might change the orientation of this loop, thereby better exposing the epitopes to anti-V3 antibodies (Figure 4). Examination of the sequences in the C3 region also revealed several changes (Figure 5c).

Of the four patients (ITM60, NYU104, 3506 and ITM27) whose sequential viruses exhibited increased sensitivity to neutralization by anti-V3 mAbs, those from patient ITM27 exhibited the weakest increase in neutralization sensitivity (Figure 2h). The sequence of the core epitope from patient ITM27's last visit was different from that of the virus obtained from the patient's first visit (Figure 4). Furthermore, the virus collected at the last time point visit from ITM27 exhibited the greatest sequence change (n = 3) in the V3 loop when compared to the virus from the first visit. These changes occurred either within the region of the core epitope (Figure 4a) or both within and near the epitope (Figure 4b-e). The three changes occurred mainly around the crown of the V3 loop and included a change from histidine (H) to proline (P) at position 13, a change from alanine (A) to glycine (G) at position 15, and a change from glycine (G) to arginine (R) at position 17. Thus, the APGS motif at the tip of the V3 loop was changed to a GPRS motif. This could be the reason why ITM27 late virus became sensitive to anti-V3 mAbs. Outside the region of the core epitope, the V3 loop sequences of the viruses from the first and last visits were identical. Of note is that in the V3 loops of the sequential viruses, even when the core epitope was present in the late time point virus, other mutations had occurred outside this core epitope (Figure 4b). The mutations in the C2 and C3 regions (data not shown and Figure 5d), might have helped in unfolding and exposing the epitopes in the V3 region which these antibodies target.

### V2 epitopes

**Figure 6:**
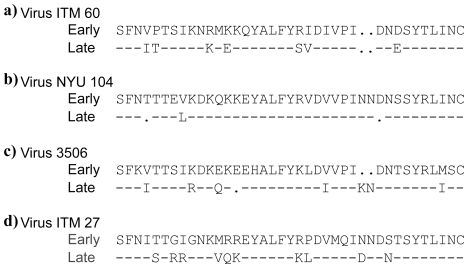
V2 region sequences of the sequential viruses. The sequences shown represent the consensus sequence from 3–11 clones. The sequences were derived from the same aliquot of virus culture supernatant that was used in the neutralization assay.

The V2 sequences of the sequential viruses from patient ITM60 and NYU104 that evolved increase sensitivity to anti-V2 mAbs were aligned and changes in the entire V2 loop sequence were examined. Several changes in the V2 sequence from the late time point virus as compared to early time point virus were identified. Unlike the constant nature of the V3 loop sequences described above, more changes were found to occur in the V2 region for the late time point virus (Figure 6). These changes in the sequence of the late time point viruses from patient ITM60 and NYU104 may well expose the epitope of anti-V2 mAbs and increase neutralization.

### CD4bd epitope (targeted by mAb IgG1b12)

We examined the changes in the amino acid sequence critical for IgG1b12 binding in sequential viruses obtained from patients ITM60, NYU104 and 3506. We found that although there were changes in the in the C3 region sequence, the amino acids critical for IgG1b12 binding had only one change for the late time point virus from patient 3506 that included a change from threonine (T) to alanine (A) at position 44 (Figure 5). No changes in amino acids critical for these anti-CD4bd mAbs were observed in both time point viruses from patient ITM60 and NYU104.

## Discussion

Our present study demonstrates that both subtype B and CRF02_AG viruses in some HIV-1 chronically-infected individuals evolve to become more sensitive to neutralization by antibodies directed at epitopes in the V2, V3 and CD4bd (Figures 2 and 3). These data provide a clear indication that snapshots of mAb neutralization capacities taken in cross sectional studies are inadequate to define the neutralization spectrum of mAb neutralization with primary HIV-1 isolates. Therefore, longitudinal studies of neutralization capacities are needed to determine important neutralization-sensitive epitopes and to better identify immunogenic regions that will be suitable for a vaccine that prevents infection by all HIV-1 strains.

Studies that have examined virus evolution and autologous neutralization in HIV-1 infected patients during acute and chronic infection have demonstrated that over time, viruses in these individuals undergo diversification and escape from neutralization [6], [8]. A potent vaccine should, by definition, induce “heterologous neutralizing” antibodies, as the infecting HIV-1 virion will differ from the vaccine strain. In recent studies, we examined the neutralization sensitivities of sequential HIV-1 subtype B strains in chronically infected patients to heterologous HIV-1 plasma samples [23]. The studies clearly demonstrated that while sequential HIV-1 viruses from some patients maintain their neutralization sensitivity to antibodies in heterologous HIV-1 plasma, as was the case for patient ITM39, other patients' viruses evolve to escape neutralization, as was the case for ITM27 [23]. While these studies with heterologous HIV-1 plasma revealed for the first time that viruses can evolve to become more sensitive to neutralization, it remained unclear which antibodies were specifically responsible for these phenomenon, due to the polyclonal nature of antibodies in plasma. Our present study demonstrates that indeed, viruses in some HIV-1 chronically infected individuals evolve increased neutralization sensitivity to antibodies directed at well defined viral epitopes. In particular, anti-V3, anti-V2, and anti-CD4bd mAbs exhibited increased neutralization capacity to viruses collected from two patients, ITM60 and NYU104, collected 31 and 29 months, respectively after initial virus that was resistant to neutralization by these same antibodies. This is the first report clearly demonstrating that as viruses evolve in their hosts, mutations occur that render them more susceptible to neutralization by specific mAbs. The possibility therefore exists that the antibodies in heterologous HIV-1 plasma samples that exhibited increased neutralization capacity to the sequential virus from this patient could in part be attributed to anti-V2, anti-V3 and anti-CD4bd antibodies. However, the pattern of neutralization sensitivity for ITM27 with the heterologous plasma and the anti-V3 mAbs may be attributed to the lack of such antibodies in the plasma.

In general, HIV-1 primary isolates are often resistant to neutralization by plasma from HIV-1 infected patients and by many anti-HIV-1 mAbs. Of the mAbs tested in this study, the anti-V3 and anti-CD4bd mAbs have been documented in other studies to neutralize primary isolates at concentrations similar to those seen in our study with the later time point viruses from the patients [24], [25]. As shown in Table 1, the IC_50_ concentrations needed to yield 50% neutralization were low (0.3–2 µg/ml), in particular, for the anti-V3 mAbs for the late time point virus from patient ITM60, while higher concentrations of the same mAbs were needed to neutralize the later time point viruses from patient NYU104 (4.4–6.1 µg/ml) and 3506 (1.5–35.5 µg/ml). Such variations in mAb concentrations needed to achieve 50% neutralization for different viruses have also been reported for these and other mAbs in other studies [24], [25]. These differences could be due to differences in the level of exposure of mAb epitopes on different viruses. For example, the V3 loop is better exposed on virus SF162 and this virus is much more sensitive to anti-V3 antibodies than other viruses whose V3 loops are less well exposed [26]. As such viruses with a better exposed epitope will possibly need a lower antibody concentration to achieve 50% neutralization than viruses whose V3 loops are less well exposed. In addition, several other factors such as specific mutations within and outside the epitope as well as folding and glycosylation patterns can contribute to differences in neutralization sensitivities requiring different mAb concentraions to achieve 50% neutralization [4], [5], [27]. These factors are discussed below with respect to the various mAbs used in this study and the mutational patterns observed based on sequence information.

The V3 loop sequences as well as the amino acids critical for mAb IgG1b12 binding remained relatively constant for the sequential viruses; yet, the viruses became more sensitive to neutralization. This suggests that changes outside the core epitope contribute to increased neutralization sensitivity. Because several changes were identified outside the core epitopes, especially in C3 region, the possibility exists that these mutations may have caused changes in the structural conformation exposing the V3 loop and increasing its accessibility to the antibodies. In addition to changes that occurred outside the core epitope, which might have resulted in better exposure and increased neutralization sensitivity, point mutations (Figure 4) might have also contributed to increased neutralization sensitivity. For example, in the case of patient ITM27, the early time point virus lacked G (glycine) at position 15 and R (arginine) at position 17, which are critical for the V3 loop β-turn. The R (arginine) at position 18 for mAbs 391/95-D, 447-52D and 694/98-D, and the G (glycine) at position 15 for mAbs 1006-15D, 2219, 2442 and 2191, are very critical for binding [2], [14], [28], [29]. The late virus for patient ITM27 has a G (glycine) at position 15 and an R (arginine) at position 18. This may have contributed in part to the increased neutralization sensitivity seen with the late time point virus from patient ITM27. However, as this virus was resistant to polyclonal antibodies in plasma, it is possible that Abs that neutralize the virus were absent in the plasma samples examined in our previous studies [23]. The V2 and V3 regions interact with each other and form a complex epitope [30], [31]. Several changes were identified in the V2 region. There is a possibility that mutations in V2 region may affect the exposure of V3 epitope and that might have contributed to increased neutralization sensitivity.

The late time point virus of NYU104 evolved increased neutralization sensitivity to the anti-carbohydrate mAb, 2G12. The 2G12 epitope is centered on the high-mannose and/or hybrid glycans of asparagines residues 295, 332, and 392, with peripheral glycans from 386 and 448 on either flank [32]. These residues are present on C2, C3, C4 and V4 region. Examination of these residues found all five N-linked glycosylation sites in both the early and late time point virus (data not shown). However, several other changes were identified in C2, C3, C4, and V4 regions from the early to the late time point virus isolate. Therefore, though the epitope required for 2G12 binding was present in the initial virus, its resistance to neutralization could be due to masking of the epitope through certain amino acid changes that probably caused folding patterns. However, we can only hypothesize that the changes in the sequences we observed around the vicinity of the epitope probably caused an increased accessibility to the antibody through better exposure resulting in increased neutralization. The data presented on the mutational patterns in figures 4, 5, 6 now provides the opportunity for studies that would identify and distinguish mutations that contribute to increased neutralization sensitivity or escape.

Viral evolution toward or away from neutralization sensitivity is likely to be an ongoing process. It now remains to be seen whether these viruses that evolve increase neutralization sensitivity will continue to be sensitive or will become resistant over time. This will require studies to examine sequential viruses from several time points from such individuals. Furthermore, our studies have examined viruses isolated from patient PBMC which might not represent the whole viral quasispecies present in the patients. However, to avoid the possibility that the neutralization sensitive viruses detected in the later time point from patients was not due to a preferential selection, adaptation and possibly infectivity differences by such variants that may be present even in the early time point, if different donor PBMCs were used for virus isolation and amplification in different donor PBMCs, the same PBMC donor pool were used in all virus isolation and amplification. Because different variants within a quasispecies can exhibit different neutralization patterns, thus, the often lack of 100% neutralization sensitivity; different variants within the patients will also need to be examined for their neutralization sensitivity over time and the proportion of variants that are susceptible to neutralization be determined.

It is important to begin to understand whether viruses that evolve increased neutralization sensitivity to heterologous antibodies also induce neutralizing antibodies in their hosts that would neutralize their autologous or other heterologous viruses. In initial experiments in which we tested the sequential plasma with their contemporaneous viruses (early and late) from patient NYU104 and ITM60 whose viruses evolved increased neutralization sensitivity we observed that these viruses were resistant to neutralization by their autologous and contemporaneous plasma samples (data not show). The results of our studies therefore reveal that while viruses mutate to escape neutralization by autologous antibodies [6], [7], some do evolve increased neutralization sensitivity to heterologous antibodies as demonstrated in our previous studies with heterologous plasma [23] and in the present study with mAbs. These results therefore, suggest that mutations that occur and lead to escape from neutralization by autologous antibodies may be different from those that lead to increased neutralization sensitivity by heterologous antibodies. Though the number of subjects studied, five, may be small, the findings are important for vaccine design and raise the need for more extended studies.

In conclusion, the results of this study are counter intuitive revealing that viruses in patients can evolve increase neutralization sensitivity. However, the antibodies tested are heterologous to the viruses, which should be the basis for a protective vaccine because the infecting virus will almost always be heterologous to the antibodies induced by the vaccine strain. Understanding how viruses evolve and how their evolution affects their sensitivity to different antibodies should improve our knowledge of the relationship between genetic evolution, immunity, and vaccine design.

## Materials and Methods

### HIV-1 infected patients and sequential virus isolation

The sequential viruses tested were isolated from the PBMCs of four HIV-1 subtype B and one CRF02_AG chronically infected drug naïve patients. Three of the four HIV-1 subtype B infected patients (ITM60, ITM27 and ITM39) from whom sequential viruses were obtained have been described in our previous studies [23]. Briefly, these patients were followed at the Institute of Tropical Medicine (ITM) in Antwerp; they were drug naïve and remained asymptomatic during a three-year study period. PBMCs from these patients were obtained by Ficol-Hypaque density gradient centrifugation; they were kept in liquid nitrogen in Antwerp and shipped in dry ice to our lab in New York for the studies described here. PBMCs from the two other subjects, 3506 (subtype B) and NYU104 were similarly obtained by Ficol-Hypaque as was for the three subtype B infected subjects from Antwerp described above. The two sequential viruses from patients ITM60, ITM27 and ITM39 were isolated at 31, 36 and 26 months intervals, respectively. The fourth HIV-1 subtype infected patient (3506) from whom two sequential viruses were obtained, was also chronically infected, drug naïve, remained asymptomatic and regularly consulted at the Veterans Affairs Medical Center, New York, New York. The two sequential viruses from patient 3506 were obtained at an 11 month interval. The fifth patient (NYU104), who was chronically infected with a CRF02_AG variant in Africa, was asymptomatic and drug naïve, and the two sequential viruses studied were obtained 29 months apart. Ethical clearances have been approved from IRB of Institute of Tropical Medicine, Antwerp, Belgium; National Ethical Committee of Cameroon and New York University Medical center.

Virus isolation and virus stock preparation from all the five patients studied were performed using two donor's PBMCs that were pooled. This approach using the same donor PBMCs was to avoid differences that may exist in virus selection, adaptation, and infectivity when using different donor PBMCs ([33], [34]). First, we isolated virus from patient by co-culturing each of 5×10^6^ patient PBMCs with of 5×10^6^ of the pooled HIV negative donor PBMCs. To prepare stocks of these viruses for the neutralization studies, 1 ml aliquot of culture supernatant containing the virus isolated from patients' PBMCs was used to infect the PBMCs of 3-day phytohemaglutinin-stimulated HIV-negative pooled donors [19], [35]. After 2–3 weeks of culture in IL-2 supplemented media, the culture supernatant from infected PBMCs was aliquoted (1 ml/tube) and stored at −80°C until use. Virus stock was titrated in TZM-bl cells as previously described, [36] and tested in neutralization assays, as described below.

### Anti-HIV-1 human mAbs

Thirteen mAbs were used to test the neutralization sensitivity of sequential viruses from the HIV-1 infected individuals. The mAbs were directed at epitopes in the V3 (391/95-D, 447-52D, 1006-15D, 2219, 694/98-D, 2442 and 2191) [2], [14], [28], [29], V2 (697-D and 2158) [17], [37], CD4bd (559/64-D, 654-D and IgG1b12) [38], [39], [40]}, and anti-carbohydrate (2G12) regions [16] of gp120. Human mAb 860-55D to parvovirus B19 [41] was used as a negative control.

### Neutralization assay

TZM-bl cell lines carrying CD4 and both CCR5 and CXCR4 were used in the neutralization assays as previously described [36]. Two fold serial dilutions of the mAbs were used starting at a concentration of 50 µg/ml up to 0.005 µg/ml and tested with 100TCID_50_ of the virus in the TZM-bl neutralization assay. Thus, culture supernatant containing PBMC derived viruses was pre-incubated with mAb for 1 hour at 37°C and added to TZM-bl cells (10,000 cells in 100 µl of growth medium containing 25 µg/ml DEAE dextran and 1 µM indinavir) in a 96-well plate. The plates were incubated for 2 days after which 150 µl of supernatant was removed and 50 µl of Bright Glo (Bright-Glo luciferase Assay system, Promega, Madison, Wisconsin) was added and incubated for 2 min at room temperature. Following this incubation period, 80 µl of the mixture was transferred to a 96 well black plate and luminescence was measured using a Lumimark Plus System microplate reader (Bio-Rad Laboratories, Hercules, CA). Reduction of infectivity was determined by comparing the relative luminescence units (RLU) in the presence of anti-HIV-1 mAbs and negative control wells in which the viruses were mixed with the human mAb 860-55D to parvovirus B19. All experiments were performed in triplicate wells. The average of the neutralization results from the triplicate wells for each virus/mAb test combination was expressed as percent neutralization. All the neutralization assays were repeated two-three times and the average of the independent neutralization assays was taken to determine the neutralization sensitivities of the various tested mAbs.

### RNA extraction and RT-PCR

Viral RNA was extracted from virus culture supernatant (the same aliquot used for neutralization) as described by Boom et al, [42] followed by reverse transcription using Oligo dT primer (Promega Corporation, Madison, WI) and AMV reverse transcriptase (Access RT-PCR system; Promega Corporation, Madison, WI). Viral RNA was heated at 70°C for 10 minutes with the Oligo dT primer and immediately chilled on ice prior to the addition of the enzyme mixture, followed by one cycle of 48°C for 1 hour. The Expand Long Template PCR System (Roche, Mannheim, Germany) was used for the first-round and second-round PCR amplification of the envelope gp160 gene according to the instructions given by the manufacturer. Amplification was performed with outer primers R8005B (forward) 5′ GCCCTGGAAGCATCCAGGAAGTCAGCCT 3′ (HXB2 location 5857–5884) and ENV N (reverse) 5′ CTGCCAATCAGGGAAGTAGCCTTGTGT 3′ (HXB2 location 9171–9145), and with inner primers R8003B (forward) 5′ AAAAGGCTTAGGCATCTCTATGGCAGGAAGAAGCG 3′ (HXB2 location 5950–5985) and R8004B (reverse) 5′ ATATGTCGACCTCGAGATACTGCTCCCACCCCTTCTGCTACTG 3′ (HXB2 location 8912–8870). The amplification protocol for the first- round PCR was one cycle at 94°C for 2 minutes, followed by 30 cycles at 94°C for 10 seconds, 55°C for 30 seconds and 68°C for 8 minutes, ending with a single extension cycle at 68°C for 10 minutes. For the second-round PCR, the protocol started with one cycle at 94°C for 2 minutes, followed by 10 cycles at 94°C for 10 seconds, 60°C for 30 seconds and 68°C for 8 minutes; it continued with an additional 20 cycles at 94°C for 15 seconds, 62°C for 35 seconds and 68°C for 8 minutes, ending with a single extension cycle at 68°C for 10 minutes.

### Cloning and Sequencing

PCR products were cloned into the Topo TA cloning vector and plasmids were transformed into chemically competent *E. coli* cells according to manufacturer's recommendations (Invitrogen, Carlsbad, CA). Between 3 and 10 colonies selected by Kanamycin (Kan) resistance and blue-white screening were grown in 3 ml of LB+Kan broth overnight. Plasmids were purified using the QIAprep Spin Miniprep kit (Qiagen Inc, Valencia, CA), and analyzed by PCR to determine insert size. Positive clones were sequenced at the 5′ and 3′ ends with the universal T3 and T7 primers. Primer walking was performed to obtain the full sequence. Sequences were assembled using the Pregap and Gap programs from the Staden software package [43]. Sequences were assessed for nonsense mutations using the Gene Cutter program from the Los Alamos HIV Sequence database website (http://hiv-web.lanl.gov/content/sequence/gene-cutter/cutter.html).
